# Reduced risk for hospitalization due to hyponatraemia in lithium treated patients: A Swedish population-based case–control study

**DOI:** 10.1177/0269881120937597

**Published:** 2020-07-20

**Authors:** Henrik Falhammar, Jakob Skov, Jan Calissendorff, Jonatan D Lindh, Buster Mannheimer

**Affiliations:** 1 Department of Molecular Medicine and Surgery, Karolinska Institutet, Stockholm, Sweden; 2 Department of Endocrinology, Metabolism and Diabetes, Karolinska University Hospital, Stockholm, Sweden; 3 Department of Medicine, Karlstad Central Hospital, Karlstad, Sweden; 4 Department of Laboratory Medicine, Division of Clinical Pharmacology, Karolinska University Hospital Huddinge, Karolinska Institutet, Stockholm, Sweden; 5 Department of Clinical Science and Education, Södersjukhuset, Karolinska Institutet, Stockholm, Sweden

**Keywords:** Hospitalization, hyponatraemia, SIADH, adverse reaction

## Abstract

**Background:**

Many drugs used in psychiatry have been reported to cause hyponatraemia. However, lithium may be an exception due to its potential for causing nephrogenic diabetes insipidus, but clinical data are largely absent. The objective of this investigation was to study the association between lithium therapy and hospitalization due to hyponatraemia.

**Methods:**

This study was a register-based case–control investigation of the general Swedish population. Patients hospitalized with a principal diagnosis of hyponatraemia (*n*=11,213) were compared with matched controls (*n*=44,801). Analyses using multivariable logistic regression adjusting for co-medication, diseases, previous hospitalizations and socioeconomic factors were deployed to calculate the association between severe hyponatraemia and the use of lithium. Additionally, newly initiated (⩽90 days) and ongoing lithium therapy was studied separately.

**Results:**

Compared with controls, the unadjusted odds ratio (OR) (95% confidence interval (CI)) for hospitalization due to hyponatraemia was 1.07 (0.70–1.59) for lithium. However, after adjustment for confounding factors the risk was reduced (adjusted OR: 0.53 (0.31–0.87)). Newly initiated lithium therapy was not significantly associated with hyponatraemia (adjusted OR 0.73 (0.35–5.38)). In contrast, for ongoing therapy the corresponding adjusted OR was significantly reduced (adjusted OR: 0.52 (0.30–0.87)).

**Conclusions:**

A marked inverse association was found between ongoing lithium therapy and hospitalization due to hyponatraemia.

## Introduction

Electrolyte disturbances are frequent, with up to 30% of hospitalized patients having hyponatraemia (Al Alawi et al., 2018; Upadhyay et al., 2006). Symptoms of hyponatraemia can be anything from mild and vague, such as lethargy, agitation and confusion, to critical, such as seizures, coma and death (Nigro et al., 2015; Renneboog et al., 2006; Spasovski et al., 2014; Verbalis et al., 2013). Hyponatraemia can be classified corresponding to the serum sodium concentration: 130–135 mmol/L defining mild, 125–129 mmol/L moderate and <125 mmol/L profound hyponatraemia (Spasovski et al., 2014). However, the clinical impact of hyponatraemia is largely dependent on its duration, with severe symptoms manifesting primarily in patients with a rapid onset (<48 h) (Spasovski et al., 2014). The single most common cause of hyponatraemia is thiazide diuretics, but many drugs common in psychiatric care, such as drugs for depression, drugs for relapse prevention and drugs for psychosis, can cause hyponatraemia requiring hospitalization by inducing syndrome of inappropriate antidiuretic hormone secretion (SIADH), increased thirst, or both (Berghuis et al., 2017; Besi et al., 2015; Coupland et al., 2011; Falhammar et al., 2018, 2019b; Farmand et al., 2018; Leth-Moller et al., 2016; Liamis et al., 2008). However, there is one drug used in psychiatry that may have an opposite effect and reduce the risk for hyponatraemia, namely lithium.

Lithium, an alkali metal present in low concentrations in human tissue, has the lowest molecular weight of all metals in the periodic system. Due to its strong relapse-preventive effect, lithium has been used as a first line therapy for bipolar disorders for more than half a century, but it is also used in recurrent depression, schizoaffective disorder, alcoholism and cluster headaches (Erden et al., 2013; Timmer and Sands, 1999).

Nephrogenic diabetes insipidus is a common complication of lithium treatment, with an estimated prevalence of 50–70% in long-term users (Bendz et al., 1994, 1996; van Melick et al., 2008). The effect of lithium therapy is accumulative, with degree of urine concentration deficit correlating with the duration of therapy (Schoot et al., 2020), with urinary concentrating capacity in individuals on long-term treatment decreased by approximately 15% compared with controls (McKnight et al., 2012). The blunted response to antidiuretic hormone (ADH) in nephrogenic diabetes insipidus secondary to lithium-treatment reduces the effect of SIADH, and high dose lithium-therapy has historically been used to treat hyponatraemia secondary to this condition (White and Fetner, 1975). Whether lithium therapy in a psychiatric context reduces the risk of severe hyponatraemia is still unknown.

The aim of this study was therefore to investigate the association between treatment with lithium and hospitalization due to hyponatraemia. Furthermore, we aimed to separate newly initiated therapy from ongoing use to study whether there was any time-dependent association.

## Methods

This was a retrospective case–control study of the Swedish general population. To identify the principal cause of the admission, the principal discharge diagnosis of each patient was utilized. All admissions and outpatient visits are coded by the attending physician in Sweden using the *International Classification of Diseases* codes, 10th Revision (ICD10). Cases were defined as adult patients (⩾18 years) hospitalized with a first-ever (defined as not occurring since 1 January 1997) principal ICD10 code of E87.1 (hyponatraemia) or E22.2 (SIADH) in The National Patient Register (NPR) from 1 October 2005 to 31 December 2014. Pseudohyponatraemia would most likely not be diagnosed with one of these ICD10 codes but instead with the diagnosis of the cause of pseudohyponatraemia, for example, multiple myeloma and other monoclonal gammopathies. Four age-, sex- and municipality-matched controls with no previous diagnosis of hyponatraemia (since 1 January 1997) per case were randomly identified from the Total Population Register. This process has been described in detail elsewhere (Falhammar et al., 2019a).

All variables used in the multiple logistic regression analysis are depicted in Table 1. To find potential confounders for hyponatraemia, ICD10 codes, Anatomical Therapeutic Chemical codes and parameters from the Longitudinal Integration Database for Health Insurance and Labor Market Studies (LISA)-register were employed (Farmand et al., 2018). Exposure to lithium was defined as a documented dispensation within 90 days prior to the index date, that is, the date of hospitalization due to hyponatraemia. The index date for hospitalization was used when identifying the matched controls. Almost all drugs for continuous use are dispensed every 90 days in Sweden (Farmand et al., 2018), and this was used to identify ongoing use. Comorbidities were controlled for since 1 January 1997 to the index date. Infectious diseases were the only exemption and were controlled for within 90 days before the index date (Table 1). Newly initiated lithium use was defined as treatment commenced within 90 days prior to the index date and at least 12 months of no exposure preceding that. The definition of ongoing lithium use also required one or more dispensations in the period 91 to 454 days preceding the index date.

**Table 1.** Variables included in the multiple logistic regression analysis and their definition.

Variables	Codes
	**ATC codes beginning with:**
**Drugs of primary interest**	
Lithium	N05AN
**Antiepileptic drugs**	
Carbamazepine	N03AF01
Oxcarbazepine	N03AF02
Phenytoin	N03AB02
Valproate	N03AG01
Lamotrigine	N03AX09
Levetiracetam	N03AX14
Gabapentin	N03AX12
**Diuretics and drugs acting on the renin–angiotensin system**	
Furosemide	C03C
Thiazides	C03A, C09BA, C09DA, C03EA
Agents acting on the renin–angiotensin system	C09
**Antibiotics**	
Fluoroquinolones	J01MA
Macrolides	J01FA
Trimethoprim sulfamethoxazole	J01EE
**Drugs for depression**	
Serotonin	N06AB
Serotonin, norepinephrine, dopamine	N06AA
Other drugs for depression	N06AX
**Other drugs**	
Amiodarone	C01BD01
Desmopressin	H01BA02
Proton pump inhibitors	A02BC, A02BD06
Drugs for psychosis (excluding lithium)	N05A excluding N05AN
NSAIDs	M01AA, M01AB, M01AC, M01AE, M01AG, M01AH, M01AX01, N02AJ08, N02AJ19
	**ICD10 codes beginning with:**
**Renal diseases**	
Renal insufficiency	N17-19, procedure codes DR016, DR024, KAS00, KAS10, KAS20
**Infections**	
Sepsis	A41
Pneumonia	J18
Meningitis	G00–G07
**Heart and vascular diseases**	
Ischaemic heart disease	I20–25
Congestive heart failure	I50
Cerebrovascular diseases	I60–64, I69
**Gastrointestinal diseases**	
Pancreatic disease	K85, K860-1
Inflammatory bowel disease	K50–51
Liver diseases	K70–77 procedure codes JJB, JJC
**Other diseases**	
Hypothyroidism	E03, E06.3
Malnutrition	E43.9, E41.9
COPD	J44
Pulmonary embolism	I26
Malignancy	C
	**Combination of ATC and ICD10 codes, each beginning with:**
Alcoholism	**ATC:** N07BB03, N07BB04, N07BB01, N07BB05, N07BB **ICD10:** E244, F10, G312, G621, G721, I426, K292, K70, K860, O354, P043, Q860, T51, Y90–91, Z502, Z714
Adrenal insufficiency	**ATC:** H02AA, H01BA **ICD10**: E27.1, E27.2, E27.3, E27.4, E25
Diabetes mellitus	**ATC:** A10 **ICD10:** E10–E14
**Socioeconomic factors**	
Education	Increasing levels of education from 1 to 6, continuous variable
Income	Income in Swedish krona during one year, continuous variable
Unemployment	Number of days, continuous variable
**Proxy for frailty**	
Drug use	Number of dispensed drugs 90 days prior to index date, categorized into <4, 4–7, 8–12 and >12 drugs
Duration of hospitalization	⩾3 days

ATC: Anatomical Therapeutic Chemical; COPD: chronic obstructive pulmonary disease; ICD: International Classification of Diseases; NSAID: non-steroidal anti-inflammatory drug

Taking advantage of the unique Swedish personal identification number, linkage between the population-based registers was performed. The NPR, The Swedish Prescribed Drug Register (SPDR) and the LISA register were utilized (Falhammar et al., 2015, 2017; Wettermark et al., 2007). In Sweden, all discharge diagnoses since 1997 and all prescriptions dispensed since 1 July 2005 can be found in the NPR and SPDR, respectively. To adjust for socioeconomic variables, the LISA register was utilized. The Regional Ethical Review Board in Stockholm (2015/2270-31/2) approved the study and, due to its retrospective epidemiological design, formal consent was waived.

### Statistical analysis

The associations between admission due to hyponatraemia and lithium use were scrutinized by means of univariable and multivariable logistic regression. The associations between lithium therapy and hyponatraemia requiring hospitalization in cases and controls were reported as unadjusted and adjusted (for potential confounders) odds ratios (ORs), with 95% confidence intervals (95% CIs). *p*-values <0.05 were considered statistically significant. For all analyses, R version 3.3.2 was used.

## Results

Hyponatraemia as a principal diagnosis was recorded in 11,213 adult individuals and 44,801 matched controls were identified. Most were females (65%) and the median age was 76 years (range 18–103). Table 2 presents a selection of comorbidities and use of lithium at baseline (index date). The most frequent comorbidities were malignancy, ischaemic heart disease, diabetes, congestive heart failure, cerebrovascular disease and alcoholism. In total, 0.25% of the cases and controls had recently been dispensed lithium.

**Table 2.** Medical characteristics (selection of items from Table 1) in addition to lithium use among cases (hospitalized with a principal diagnosis of hyponatraemia) and controls at index date.

	Number of total cases (*n*=11,213)	Number of total controls (*n*=44,801)
**Diagnosis**		
Malignancy	3096 (27.6%)***	9149 (20.4%)
Ischaemic heart disease	2186 (19.5%)***	6290 (14.0%)
Diabetes mellitus	1939 (17.3%)***	5277 (11.7%)
Alcoholism	1764 (15.7%)***	833 (1.9%)
Congestive heart failure	1453 (13.0%)***	3533 (7.9%)
Cerebrovascular disease	1448 (12.9%)***	3533 (7.9%)
COPD	1125 (10.0%)***	1576 (3.5%)
Hypothyroidism	1139 (10.2%)***	1994 (4.5%)
Adrenal insufficiency	631 (5.6%)***	352 (0.8%)
Renal disease	489 (4.4%)***	888 (2.0%)
Liver disease	421 (3.8%)***	332 (0.7%)
Pancreatic disease	252 (2.2%)***	395 (0.9%)
IBD	221 (2.0%)***	444 (0.1%)
**Medications**		
Drugs for depression	2817 (25.1%)***	5745 (12.8%)
Drugs for psychosis	772 (6.9%)***	1096 (2.4%)
Antiepileptic drugs	1061 (9.5%)***	1128 (2.5%)
Furosemide	1735 (15.5%)***	5487 (12.2%)
Thiazide diuretics	4364 (38.9%)***	6103 (13.6%)
**Proxy for frailty**		
Number of dispensed drugs 90 days prior to index date		
<4 drugs	2215 (19.8%)***	22892 (51.1%)
4–7 drugs	3421 (30.5%)**	12,967 (28.9%)
8–12 drugs	3558 (31.7%)***	7010 (15.6%)
>12 drugs	2019 (18.0%)***	1932 (4.3%)
Number of hospitalizations ⩾3 days duration	4852 (43.2%)***	9477 (21.2%)
**Lithium medication**		
Total	29 (0.26%)	108 (0.24%)
Newly initiated	1 (0.009%)	5 (0.01%)
Ongoing use	28 (0.2%)	103 (0.2%)

** *p*<0.01; ***p<0.001 compared with controls.

COPD: chronic obstructive pulmonary disease; IBD: inflammatory bowel disease

**Figure 1.**
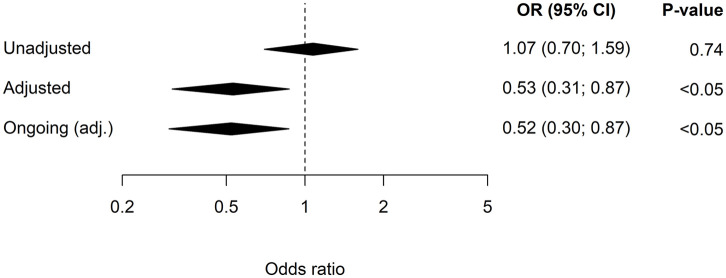
The unadjusted and adjusted (adjusted for all the confounding factors in Table 1) odds ratios (ORs), including 95% confidence intervals (95% CIs) for hospitalization due to hyponatraemia in patients on lithium. The ongoing treatment (⩾90 days) is adjusted (adj.) OR (95% CI).

The association between exposure to lithium and hospitalization due to hyponatraemia is depicted in Figure 1. Compared with controls, the unadjusted OR (95% CI) for hospitalization due to hyponatraemia was 1.07 (0.70–1.59) for lithium. However, after adjustment for confounding factors the risk decreased and was lower compared with controls (adjusted OR: 0.53 (0.31–0.87)).

The effects of newly initiated lithium use versus ongoing therapy (adjusted ORs) were also calculated. Newly initiated treatment with lithium was rare in both cases and controls (*n*=1 and *n*=5, respectively) and did not demonstrate any significant association with hospitalization for hyponatraemia (adjusted OR: 0.73 (0.35–5.38)). In contrast, in patients with ongoing use, the adjusted ORs showed a significantly reduced risk for hyponatraemia among lithium-treated patients (adjusted OR 0.52 (0.30–0.87)) (Figure 1).

## Discussion

This is the first population-based study reporting on lithium therapy and hyponatraemia. The association with hyponatraemia requiring hospitalization was halved in individuals treated with lithium.

The mechanism of lithium-induced protection against hyponatraemia is most likely its nephrogenic diabetes insipidus potential (McKnight et al., 2012). In cell models, lithium has been shown to downregulate aquaporin-2 transcription and to cause nephrogenic diabetes insipidus independent of adenylyl cyclase activity (Li et al., 2006). This results in aquaresis without loss of sodium or other electrolytes, manifesting as weight loss and hypernatraemia if fluid intake is insufficient to compensate for urinary losses. Development of nephrogenic diabetes insipidus takes time, usually many years (Bockenhauer and Bichet, 2015), most likely explaining why the reduced risk of severe hyponatraemia is apparent only in individuals with ongoing lithium therapy. However, due to the small number of individuals with newly initiated treatment, the conclusion is uncertain in this regard. In contrast, thiazide diuretics, which also increase diuresis, are linked to hyponatraemia, partly due to increased urinary sodium losses, demonstrating that increased urinary output can have opposite effects on plasma sodium concentrations.

Interestingly, glucose-lowering medications also reduce the risk for hospitalization due to hyponatraemia (Falhammar et al., 2020). Whether this is due to the glucose-lowering medications by themselves or the underlying diabetes disorders is not clear but the mechanism may involve osmotic diuresis caused by glucosuria counteracting the water retention associated with SIADH, not unlike the effects in nephrogenic diabetes insipidus secondary to lithium.

This present study has both strengths and limitations. The major strength is the population-based design including all individuals hospitalized with a principal diagnosis of hyponatraemia in the entire country of Sweden during almost a decade. The main limitation, though, is that plasma sodium concentrations were not available. On the other hand, since we considered only patients with a principal diagnosis of hyponatraemia, we ascertained that only patients with clinically relevant hyponatraemia were included. This is a major strength compared with studies including patients with hyponatraemia as a secondary diagnosis, diagnoses made in the secondary care (Coupland et al., 2011) or patients with a mild to moderate hyponatraemia regardless of symptoms (Leth-Moller et al., 2016). To further strengthen our study approach, our prior validation of the principal diagnosis of hyponatraemia found that 89% had been hospitalized primarily due to symptoms of hyponatraemia and the mean plasma sodium concentration was 121 mmol/L (Farmand et al., 2018). Furthermore, the vast majority (77%) had a sodium concentration less than 125 mmol/L (Farmand et al., 2018), that is, profound hyponatraemia (Spasovski et al., 2014), providing further evidence of the clinical relevance of the study design. However, there is a risk of confounding in observational studies and even though we did adjust for a large number of comorbidities and medications there is always the risk of residual confounding. A large effect of adjustment, as we see in the present study, may indicate an increased risk of residual confounding. Another possible confounder/limitation is that patients who are prescribed lithium undergo regular blood tests of renal function. In theory, low plasma sodium concentrations would therefore be detected more often in lithium treated patients than in controls. If the causal factor was identified and remedied without hospitalization, this would confound our observations in a negative direction. On the other hand, if it resulted in hospitalization for correction of hyponatraemia, it could potentially confound our results in a positive direction. Moreover, nephrogenic diabetes insipidus is related not only to the duration of lithium treatment, but also to the dosage of lithium. It is common to start with a lower dose and then titrate up according to the serum levels of lithium. However, we were unable to evaluate cumulative effects of lithium treatment as we did not have access to information on lithium doses or serum lithium concentrations. It should also be noted that the number of patients using lithium, both cases and controls, was limited (*n*=29 and *n*=108, respectively), resulting in a rather large CI. The result should therefore be interpreted with caution and confirmed in larger studies.

The current study has some important clinical implications. In a patient requiring psychiatric medications such as drugs for depression, drugs for psychosis or antiepileptic drugs (as drugs for relapse prevention) and with a history of hyponatraemia or increased risk of hyponatraemia, lithium therapy, if adequate, could be an alternative, as it is unlikely to induce or exacerbate hyponatraemia. Moreover, in a patient treated with lithium presenting with hyponatraemia, causes other than lithium are more likely to explain the onset of hyponatraemia. However, the decision to switch from another psychiatric medication to lithium should be based on the underlying psychiatric condition, not plasma sodium concentrations. Initiating lithium therapy to treat manifest hyponatraemia due to SIADH or to reduce future risk of severe hyponatraemia is not a reasonable strategy as the protective effect of lithium is dependent on high doses or on long-term use and the long-term effects on renal function may vary on an individual level. Furthermore, lithium is dangerous in overdose, thus requiring regular monitoring of serum concentrations to keep within the narrow therapeutic window between toxicity and effectiveness, and situations that predispose to sodium or volume depletion may result in lithium intoxication (Okusa and Crystal, 1994). Finally, the present report exemplifies the magnitude of post marketing surveillance to reveal hitherto unrecognized properties, both negative and positive, that may be associated with a medication and to evaluate the real-world effectiveness and safety of the drug (Mazzitello et al., 2013; Sportiello et al., 2016).

In conclusion, a marked inverse association was found between lithium therapy and hospitalization due to hyponatraemia. The mechanism is most likely mediated by lithium-induced nephrogenic diabetes insipidus. The main clinical implication of this study is that lithium treatment is an unlikely cause of hyponatraemia.
